# A Pilot Feasibility Evaluation of a Heart Rate Variability Biofeedback App to Improve Self-Care in COVID-19 Healthcare Workers

**DOI:** 10.1007/s10484-024-09621-w

**Published:** 2024-03-19

**Authors:** Janell L. Mensinger, Guy M. Weissinger, Mary Ann Cantrell, Rachel Baskin, Cerena George

**Affiliations:** 1 Department of Clinical and School Psychology, College of Psychology, Nova Southeastern University, 3301 College Ave, 1073 Maltz, Fort Lauderdale, FL 33314 USA; 2 Fitzpatrick College of Nursing, Villanova University, Villanova, PA USA

**Keywords:** Heart rate variability biofeedback, Mind–body health, Interoception, Disordered eating, Eating behavior, Healthcare workers

## Abstract

COVID-19 exacerbated burnout and mental health concerns among the healthcare workforce. Due to high work stress, demanding schedules made attuned eating behaviors a particularly challenging aspect of self-care for healthcare workers. This study aimed to examine the feasibility and acceptability of a heart rate variability biofeedback (HRVB) mobile app for improving well-being among healthcare workers reporting elevated disordered eating during COVID-19. We conducted a mixed methods pre-mid-post single-arm pilot feasibility trial (ClinicalTrials.gov NCT04921228). Deductive content analysis of participants’ commentary generated qualitative themes. Linear mixed models were used to examine changes in pre- mid- to post-assessment scores on well-being outcomes. We consented 28 healthcare workers (25/89% female; 23/82% Non-Hispanic White; 22/79% nurses) to use and evaluate an HRVB mobile app. Of these, 25/89% fully enrolled by attending the app and device training; 23/82% were engaged in all elements of the protocol. Thirteen (52%) completed at least 10 min of HRVB on two-thirds or more study days. Most participants (18/75%) reported being likely or extremely likely to continue HRVB. Common barriers to engagement were busy schedules, fatigue, and technology difficulties. However, participants felt that HRVB helped them relax and connect better to their body’s signals and experiences. Results suggested preliminary evidence of efficacy for improving interoceptive sensibility, mindful self-care, body appreciation, intuitive eating, stress, resilience, and disordered eating. HRVB has potential as a low-cost adjunct tool for enhancing well-being in healthcare workers through positively connecting to the body, especially during times of increased stress when attuned eating behavior becomes difficult to uphold.

**Supplementary Information:**

The online version contains supplementary material available at 10.1007/s10484-024-09621-w.

## Introduction

Even before COVID-19, the healthcare workforce reported substantial burnout and mental health concerns (Mousavi et al., 2017; Shanafelt et al., 2012). Worse patient safety outcomes are one notable consequence of healthcare worker burnout (Cimiotti et al., 2012; Hall et al., 2016). Multiple empirical studies conducted during COVID-19 revealed that the pandemic severely intensified distress and burnout among healthcare workers (Barello et al., 2020; Sultana et al., 2020; Vizheh et al., 2020). Compared to their non-nursing healthcare counterparts, nurses were particularly prone to suffering from the psychological impact of the COVID-19 pandemic (Batra et al., 2020; Lai et al., 2020).

The nurse’s role as “care provider” is so central to the mental and physical health and well-being of others, we often fail to acknowledge the ways in which nurses tend to neglect their own self-care needs (Mullen, 2015, 2015). In fact, Logan et al., (2023) recently found work stress to be an important negative predictor of self-care among nurses. Most nurses report that the opportunity for breaks during the workday is very rare (Linton & Koonmen, 2020; Ross et al., 2019), making it difficult to engage in an important aspect of self-care—nourishing the body using attuned eating (Hotchkiss, 2018; Nahm et al., 2012). Consistent with these findings, several studies found healthcare professionals, and nurses in particular, to be at high-risk for disordered eating, especially when job-related stressors were high (King & Arthur, 2004; Marko et al., 2022; Nicholls et al., 2017). Wong et al. (2010) reported that nearly two-thirds of a sample of nurses (N = 378) had elevated scores on a validated measure of maladaptive eating patterns.

In the general population, recent distress caused by the uncertainties of the COVID-19 pandemic were met with with considerable increases in disordered eating and full blown eating disorders across the globe (e.,g., Breiner et al., 2021; Freizinger et al., 2022; Ramalho et al., 2022; see Devoe et al., 2023 for a systematic review of the literature). Theoretical models of attunement to the body have established mindful self-care as a meaningful target of therapy for disordered eating (e.g., Cook-Cottone, 2015). Given high levels of reported psychological distress and needs among healthcare workers post-pandemic (Copel et al., 2023; Mensinger et al., 2022), the mindful self-care framework was recently studied in a sample of individuals working in the helping professions and showed reduced burnout was associated with frequent mindful self-care practices (Hotchkiss & Cook-Cottone, 2023).

Amidst COVID-19-related stressors such as staff shortages, overflowing intensive care units, and lack of adequate personal protective equipment (Emanuel et al., 2020; Livingston et al., 2020; Theorell, 2020), it became critical to study interventions to aid healthcare workers in coping with pandemic circumstances in effort to maximize resilience and prevent further exacerbation of burnout. A Cochrane review of interventions to improve the mental health and resilience of frontline healthcare workers during pandemics of the past two decades found no ‘high confidence’ interventions, underscoring a need for more research on this matter (Pollock et al., 2020).

To better understand predictors of resilience and well-being, multiple researchers have examined ‘sense of coherence’ as a relevant construct during COVID-19 (Ruiz-Frutos et al., 2021; Szovák et al., 2020). Sense of coherence is defined as the capacity to see one’s life as meaningful, comprehensible, and manageable (Antonovsky, 1987); and it was developed as part of Antonovsky’s theory of ‘salutogenesis’ (Antonovsky, 1996)—i.e., the origins of *health*—or inquiry into the processes that enable one to stay well. Colomer-Pérez et al. (2022) used a salutogenic model of health in a sample of 921 students training to be nursing assistants and found sense of coherence to be positively associated with agency to engage in self-care. Decades of research demonstrating a robust relationship between sense of coherence, lower traumatic stress, and multiples markers of well-being, including burnout and job satisfaction among nurses (Eriksson & Lindström, 2006; Masanotti et al., 2020; Schäfer et al., 2019), creates a solid foundation from which to build a strengths-based, salutogenic intervention to promote health, especially warranted during times of acute distress like the COVID-19 pandemic (Schäfer et al., 2020).

A related psychophysiological model of resilience that has also been well-studied as a factor connected to psychosocial well-being, self-regulation, and autonomic nervous system functioning is known as *cardiac* coherence (McCraty & Zayas, 2014). More specifically, Respiratory Sinus Arrythmia (RSA) is a temporal coherence between heart rate and breathing achieved when there is efficient synchronization between our physiological systems that allows for optimal modulation of emotional states, functional performance, and overall health (Bradley et al., 2010; McCraty & Zayas, 2014; Segerstrom & Nes, 2007). RSA, which reflects interactions between the sympathetic nervous system (causing heart rate to increase) and the parasympathetic nervous system (causing the slowing of heart rate) is captured by examining the beat-to-beat changes in heart rate, also known as heart rate variability (HRV) (Sevoz-Couche & Laborde, 2022; Shaffer et al., 2014). For decades researchers have studied how HRV is associated with physical and emotional states (Lee et al., 2023; Thayer et al., 2009; Zhu et al., 2019).

Higher HRV is a predictor of health and longevity (Piccirillo et al., 2001; Ponikowski et al., 1997; Zulfiqar et al., 2010) and has even been used to detect COVID-19 cases among healthcare workers in the early part of the pandemic (Hasty et al., 2021). HRV biofeedback (HRVB) is a technique that uses audio and/or visual cues derived via signals generated from blood flow and respirations detected in the body (often using photoplethysmography technology or PPG) to train people to become more attuned to the interplay of physiological markers such as the breath and heartbeat (Lehrer & Gevirtz, 2014). This mind–body connection supports people in achieving optimal HRV levels and has been found in meta-analyses to help with a wide variety of stress-related concerns, including anxiety, traumatic stress, anger, depression, athletic and artistic performance, chronic pain, and sleep problems (Goessl et al., 2017; Lehrer et al., 2020). Buchanan and Reilly (2019) successfully used HRVB to reduce distress in a sample (N = 26) of female healthcare professionals at an US academic medical center. Their research reported significant improvements in emotional, physical, and organizational stress, including anxiety and depressive symptoms, anger, resentment, fatigue, general health symptoms, and relational tensions at work after applying HeartMath® techniques for 4 to 6 weeks.

While many have focused on the physiological mechanisms through which HRVB yields health benefits (Lehrer, 2013; Lehrer & Gevirtz, 2014; McCraty & Shaffer, 2015), a recent review of the literature suggests the positive effects of HRVB on emotions may occur by improving interoceptive sensibility (Pinna & Edwards, 2020). The overarching construct of interoception is defined as our conscious capacity to integrate and adaptively respond to body-related signals such as hunger, thirst, temperature, and pain; it is a crucial survival mechanism for maintaining homeostasis (Craig, 2002). Deficits in interoception are well known to be evident in individuals with mental health concerns, especially those with eating disorders (Füstös et al., 2013; Löffler et al., 2018; Martin et al., 2019). Using HRVB for disordered eating has become a promising area of research warranting more attention (Godfrey et al., 2019; Meule et al., 2012; Scolnick et al., 2014).

### Study Aims

Given the importance of enhancing healthcare worker well-being, the purpose of the present study is to report the acceptability, usability, and early signals of efficacy for HRVB with a commercially available smartphone app in healthcare professionals reporting elevated disordered eating during the first year of the pandemic. Specifically, this mixed methods pilot feasibility trial primarily aimed to test study enrollment, intervention retention, engagement, acceptability, and usability. Qualitative and quantitative data were integrated to answer usability and intervention acceptability. The secondary aim involved testing preliminary evidence of efficacy through examining changes in disordered eating, perceived stress, resilience, interoceptive sensibility, and mindful self-care over the course of the intervention. In sum, the goal of the current study was to evaluate data gathered in preparation for developing a future definitive trial utilizing a mindfulness-based HRVB smartphone app in a mind–body intervention for disordered eating.

## Method

This study was a mixed methods pre-mid-post single arm non-randomized feasibility trial. Ethics approval was received from the Villanova University IRB and registered on ClinicalTrials.gov prior to beginning recruitment (registration identifier NCT04921228).

### Sample

Study participants were recruited from a pool of healthcare workers enrolled in the CHAMPS Registry, a survey study of the health and well-being of healthcare workers during the first year of the COVID-19 the pandemic (May 2020–Decemeber 2020). The methods and purpose of the CHAMPS Registry are described in more detail in the published protocol (Kaufmann et al., 2021) and initial findings (Copel et al., 2023; Mensinger et al., 2022). Participants invited to the HRVB study reported elevated levels of disordered eating (i.e., >2.6 on the brief Loss of Control over Eating Scale, which is considered clinically significant according the validation study by Stefano et al., 2016) as part of the CHAMPS study data collection in 2020. Participants also had to be English speaking and own a smartphone for the intervention app. We excluded participants with serious health conditions and behaviors that would significantly impact HRV readings including: a) history of heart transplant; b) current pacemaker; c) uncontrolled kidney disease; d) uncontrolled diabetes; e) heart failure; f) chronic obstructive pulmonary disease; g) use of tricyclic antidepressants above 75 mg per day; and h) use of illicit stimulants or narcotic drugs. We also excluded participants who were currently practicing HRVB or had prior experience using a mobile HRVB app. See Table 1 for the sample characteristics.

**Table 1:** Demographic and workplace characteristics of the baseline study sample who pre-enrolled in pilot study of HRV biofeedback app (N = 28)

Characteristic	*n* (%)
Healthcare occupation	
Nurse	22 (79)
Chaplain	2 (7)
Physician	1 (4)
Pharmacy Technician	1 (4)
EMT/Fire Department	1 (4)
Social Worker	1 (4)
Workplace setting	
Large metropolitan hospital	14 (50)
Suburban/regional hospital	4 (14)
Rural/community hospital	2 (7)
Long-term care facility	2 (7)
Face-to-face ambulatory/outpatient care	4 (14)
Field setting (e.g., EMT roles)	1 (4)
Other setting (e.g., telehealth)	1 (4)
Years working in the profession^a^	
More than 20 years	8 (29)
11–20 years	8 (29)
5–10 years	4 (14)
Less than 5 years	7 (25)
US Region	
Northeast	16 (57)
South	4 (14)
Midwest	5 (18)
West	3 (11)
Race/Ethnicity	
African American/Black	2 (7)
Non-Hispanic White	23 (82)
Hispanic/Latinx	2 (7)
Asian/Pacific Islander	1 (4)
Marital Status	
Married/Domestic Partnership	18 (64)
Single/Never married	8 (29)
Divorced	1 (4)
Separated	1 (4)
Age	
More than 60 years	5 (18)
45–59 years	8 (29)
30–44 years	14 (50)
Less than 30 years	1 (4)
Gender Identity	
Female	25 (89)
Male	3 (11)
Mean years working in profession *(SD),* Range	16.15 (12.33), 1–40
Mean Age, years (*SD*), Range	45.6 (11.8), 28–65

All demographic data collected during 2020 CHAMPS study

^a^Missing 1 response on years working in profession

### Study Procedures and Intervention

#### Recruitment and Consent

**Fig. 1:**
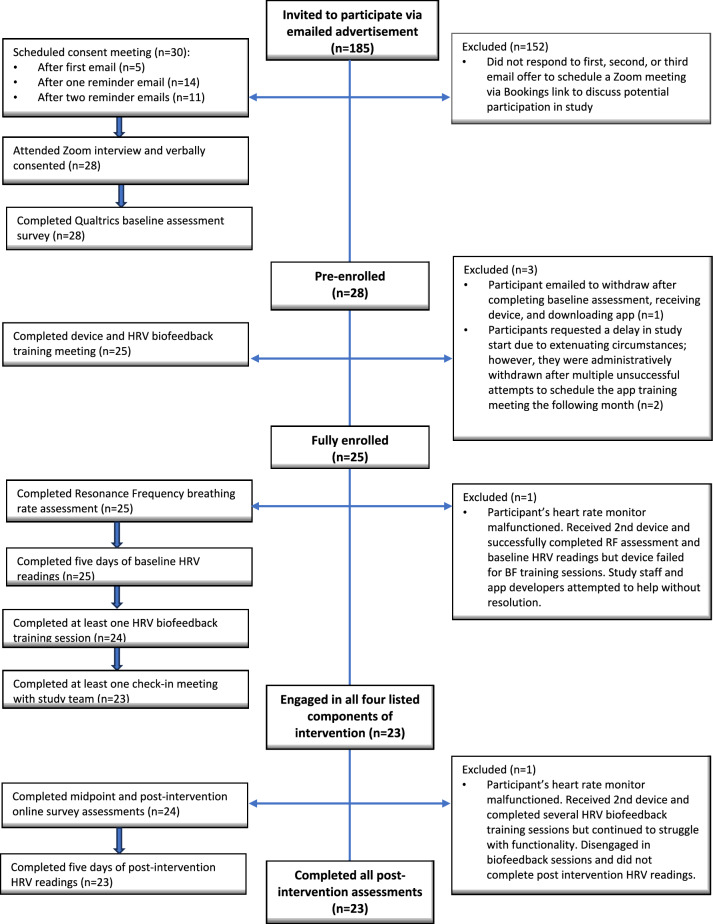
Flow of recruitment and retention through study

Figure 1 provides a flowchart of recruitment and retention throughout the study. Emails were sent to 185 participants from the CHAMPS Registry who met inclusion criteria (exclusion criteria were determined during informed consent interviews). Using the Booking app, links were included in the communication so that interested individuals could self-schedule a Zoom-based interview to review the protocol with the principal investigator (JM) and offer verbal informed consent. Thereafter, interested participants were emailed the consent document and a link to a Qualtrics baseline assessment survey. Upon completion of the baseline assessment, Qualtrics emailed a notification to the study team to mail the heart rate device to the participant’s provided address. At this time, participants were sent electronic and hard copies of instructions for downloading and using the HRVB app (OptimalHRV; available on the app store for iPhones and Androids). The app itself was also equipped with a myriad of instructional videos including background information about HRV and HRVB training.

#### App and Device Training

After receiving a postal service delivery confirmation of the heart rate device, we emailed invitations with a Bookings link to schedule a one-on-one app and device training via Zoom with the PI. The training included another review of the intervention protocol and demonstration of the app and device using screen sharing tools. Specifically, we explained that before beginning HRVB sessions, participants were required to complete 5 days of 3-min HRV readings (recorded as Root Mean Square Standard Deviation, RMSSD) to gauge baseline HRV. We requested that they do the HRV readings on consecutive days, upon awakening from a night’s rest, and explained the importance of breathing normally and not talking or moving during the HRV readings. At the end of the 2-month intervention, 5 days of HRV readings were to be repeated, ideally at the same time and in the same setting as they had completed their baseline readings. Participants were also shown how to complete an app-guided resonance frequency (RF) breathing rate assessment. Completing the 15-min RF assessment was required 1 time for the protocol. We recommended participants did the RF assessment after practicing low and slow breathing using the app and *before* beginning HRVB practice. This allowed participants to choose the appropriate breathing pacer speed in the app when doing HRVB sessions. Training meetings averaged approximately 60 to 90 min in length.

In addition, with the ultimate goal of reaching 20 min of daily HRVB after 2 weeks, we suggested beginning with 4 or 5-min HRVB sessions initially and increasing session length incrementally every few days since small and gradual change accompanied by repetition helps establish a new healthy habit (Gardner et al., 2012; Phillips-Caesar et al., 2015). Twenty-minute HRVB sessions are a well-established norm in the field and a common recommended session length in controlled studies of HRVB (Lalanza et al., 2023). In alignment with self-determination theory, we aimed to maximize autonomy through flexibility and avoiding a rigid protocol by giving no further specifics about practice goals (Ryan et al., 2021).

#### Study Check-Ins During HRV Biofeedback Training

To ensure participants had opportunities to discuss problems, barriers, and experiences with the intervention, we held 15-min weekly check-ins via Zoom or phone calls with study team members. After the app training with the PI, a team member was assigned to each participant for weekly check-ins to serve as a continual contact and coach during the study. We anticipated meeting with participants 7 to 8 times during the intervention protocol. Upon completion of each check-in, team members wrote a note to document the discussion, which covered usability issues (barriers and facilitators), efforts made to support engagement, perceptions of HRVB, and any additional feedback about the study and the app.

#### Tools to Enhance Protocol Adherence

The Outlook Bookings app was used so that participants could self-schedule with their assigned team member in accordance with their and the team member’s availability. Automated reminder messages via email (24 h in advance) and text (30 min in advance) were used throughout the study to minimize scheduling oversights and improve attendance at scheduled meetings. We also manually sent weekly motivating emails to those who were found to be struggling with engaging in HRVB (e.g., missing more than 3 consecutive days), or congratulating emails to those doing very well with HRVB practice (e.g., doing biofeedback most days of the week). Participants were sent a $75 Amazon gift card in appreciation for their time after completing the post-intervention assessment.

### Measurements

#### Demographics

Participants self-reported gender identity, age, race/ethnicity, marital status, occupation, workplace setting, years on the job, etc. when enrolling in the CHAMPS study of COVID-19 pandemic essential workers’ health and well-being. These data were merged into the feasibility trial dataset using study ID numbers.

#### Disordered Eating

Two measures were used to assess disordered eating. The Loss of Control over Eating Scale-Brief (LOCES) (Latner et al., 2014) and the Eating Disorder Examination Questionnaire-Short (EDE-Q7) (Grilo et al., 2015) are 7-item brief versions of the scales. The questionnaires provide a timeframe of the past 4 weeks/28 days. The LOCES taps into questions surrounding binge eating behaviors and specifically the sense of loss of control (e.g., “I continued to eat past the point when I wanted to stop”) and uses a 5-point Likert scale (1 = *never* to 5 = *always*). The EDE-Q7 uses a 7-point scale (0 = *no days* to 6 = *every day* or 0 = *not at all* to 6 = *markedly*) and provides a global disordered eating score assessing dietary restraint (extreme food restriction in effort to reduce weight), how weight and shape influence self-worth, and body dissatisfaction. Composite scores for both scales were derived by calculating means of the 7 items with higher scores indicative of greater disordered eating. At baseline, the Cronbach’s alpha of LOCES was 0.92 and for the EDE-Q7 alpha was 0.76.

#### Perceived Stress

We assessed perceived stress using Cohen’s 10-item version of the Perceived Stress Scale (PSS) (Cohen et al., 1983). Responses to the PSS are evaluated on a 5-point Likert scale (0 = *never* to 4 = *very often*). We derived composite scores by summing items, after reverse scoring the four positively worded questions, so that higher scores indicate greater stress. Potential scores range from 0 to 40. At baseline, Cronbach’s alpha was 0.85.

#### Resilience

Antonovsky’s revised Sense of Coherence Scale was used as a measure of resiliency (SOC-R) (Hittner, 2007; McGee et al., 2018). The SOC-R contains 13 items that assess an individual’s capacity to cope and view the events of the world and their life from multiple, balanced perspectives (e.g., “One can always find a way to cope with painful things in life,” and “I always try to see things in context”). Items are rated on a 5-point Likert scale (1 = *not at all true* to 5 = *extremely true*) and composite scores are derived by summing the items after reverse scoring the negatively worded item. Potential scores range from 13 to 65 with higher scores suggesting greater resilience. At baseline, the Cronbach’s alpha was 0.78.

#### Interoceptive Sensibility

We used the Multidimensional Assessment of Interoceptive Awareness v2 (MAIA) (Mehling et al., 2018) which was developed as a comprehensive tool to measure interoceptive sensibility (an important self-reported component of interoception), especially in the context of mind–body interventions. The revised MAIA contains 37 items rated on a 6-point Likert scale (0 = *never* to 5 = *always*). Composite scores were created by taking a mean of the items on each dimension, after reverse scoring 9 items. Given the independent nature of each dimension (Ferentzi et al., 2021; Mehling et al., 2018), we analyzed the subscales individually as follows: (1) *noticing*—awareness of body sensations, contains 4 items (baseline Cronbach’s alpha of 0.77); (2) *not distracting*—tendency to not ignore or distract oneself from sensations of pain, contains 6 items (baseline Cronbach’s alpha of 0.92); (3) *not worrying*—tendency to not worry or experience distress with sensations of pain, contains 5 items (baseline Cronbach’s alpha of 0.92); (4) *attention regulation*—ability to sustain attention to body sensations, contains 7 items (baseline Cronbach’s alpha of 0.89); (5) *emotional awareness*—awareness of the connection between body sensations and emotional states, contains 5 items (baseline Cronbach’s alpha of 0.75); (6) *self-regulation*—ability to regulate distress by attention to body sensations, contains 4 items (baseline Cronbach’s alpha of 0.81); (7) *body listening*—active listening to the body for insight, contains 3 items (baseline Cronbach’s alpha of 0.89); and (8) *trusting*—experience of one’s body as safe and trustworthy, contains 3 items (baseline Cronbach’s alpha of 0.92). Higher scores on all dimensions reflect greater interoceptive sensibility.

#### Mindful Self-Care

Multiple constructs related to self-care behaviors were assessed. First, we used the Reliance on Hunger and Satiety Cues subscale of the Intuitive Eating Scale-2 (Tylka & Kroon Van Diest, 2013). This subscale contains 6 items rated on a 5-point scale (1 = *strongly disagree* to 5 = *strongly agree*). Items are averaged to create a composite score of intuitive eating with higher scores reflecting eating with a greater reliance on hunger and satiety cues. Cronbach’s alpha was 0.78 at baseline. To measure a related but distinct component of self-care we used the 10-item Body Appreciation Scale-2 (BAS-2), which assesses a positive regard toward one’s body (Tylka & Wood-Barcalow, 2015). Items are rated on a 5-point scale (1 = *never* to 5 = *always*) and are averaged to create a composite score where higher scores show more body appreciation. Cronbach’s alpha for the BAS-2 was 0.96 at baseline. The final self-care construct was assessed with the Mindful Self-Care Scale-Brief (MSCS-B) (Hotchkiss & Cook-Cottone, 2019, 2023). The MSCS-B was developed to determine whether health promoting interventions serve to improve self-care behaviors and mindful awareness using 6 domains—*mindful relaxation, physical care, self-compassion and purpose, supportive relationships, supportive structure, and mindful awareness*. The brief version of the tool assesses the frequency over the past week of 24 mindful behaviors rated on a 5-point Likert scale (0 = *never/0 days* to 5 = *regularly/6–7 days*), several of which were very slightly modified to ensure relevance for participants in the present study (see Supplementary Table A). A valid and reliable model supporting a single dimension construct for the 6 facets of mindful self-care has been found in several studies of health professionals (Hotchkiss, 2018; Hotchkiss & Cook-Cottone, 2023). Composite scores were created by taking a mean of the 24 items after reverse scoring an item. Cronbach’s alpha for the total scale was 0.91 at baseline.

#### App Usability/Acceptability

The final questions on the post intervention survey asked participants to rate the following questions: (1) how useful they found HRV biofeedback (1 = *not at all useful* to 5 = *extremely useful*); (2) how likely they were to continue using HRV biofeedback (1 = *extremely unlikely* to 5 = *extremely likely*); and (3) how likely they were to recommend HRV biofeedback to a friend or coworker (1 = *extremely unlikely* to 5 = *extremely likely*). Given the early recognition of problems with device connectivity to the app, we added a question inquiring if connectivity problems were encountered during the study to the post intervention survey. If the participant answered yes, we asked a follow-up question about the extent to which the connectivity issues interfered with doing HRVB practice in accordance with the study protocol (1 = *never* to 5 = *always*). We also posed an optional open-ended question asking participants to describe their experience with the intervention (mid- and post-intervention surveys) and barriers and/or facilitators to using HRVB during the study (post survey only).

### Data Analysis and Feasibility Markers

Based on recommendations for adequate sample sizes for pilot feasibility studies, (Hertzog, 2008) we recruited 28 participants. A sample size of 20 is considered sufficient for pilot efficacy testing for within-subjects study designs implementing 3 repeated measurements when effect sizes are at least moderate (Hertzog, 2008). Given that many studies have determined HRVB to show significant change with medium effect sizes on related outcomes (Lehrer et al., 2020), we anticipated adequate power to detect a potential effect, even with 20% attrition.

To detect enrollment feasibility, we calculated the proportion of the individuals contacted via email who: (a) scheduled Zoom-based consent interviews, (b) attended the interview, (c) verbally consented to participate, (d) pre-enrolled, and (e) fully enrolled in the study. Pre-enrollment was defined as those who had verbally consented during the Zoom interview and subsequently completed the baseline assessment which contained documented agreement to participant. Full enrollment was defined as those who completed the required heart rate device and app training meeting. Study retention was defined using multiple phases of the protocol as outlined in Fig. 1.

Recent HRVB protocol guidelines recommend 10–20 min of at home HRVB practice on most days (Lalanza et al., 2023); thus, study engagement was defined a priori as the number of days on which *at least* 10 min of HRVB was completed. We considered the first recorded HRVB session after the participant completed the 5 pre-intervention HRV readings, no matter the length (often it was only 2 min), to mark the beginning date of the intervention. We counted any day with 10 or more minutes of HRVB practice over the following 2 months as a day the participant engaged in the protocol. HRVB sessions recorded after 2 months had passed were not counted. Our goal was for 75% of fully enrolled participants to practice ≥ 10 min of HRVB on 28 or more days (i.e., 2/3 of 42 days). This represented a ≥ 67% adherence rate after accounting for the 2-week training and titration period. We also present total minutes of HRVB practice over the course of the 2-month intervention as a supplementary engagement outcome.

App acceptability and usability was assessed by examining the proportion of participants rating the app as at least ‘very useful’. Similarly, we aimed to reach a rating of at least ‘likely’ to continue using, and a rating of at least ‘likely’ to recommend the app to peers (all 4s out of a 5-point scale). To provide a more holistic understanding of participants’ experience of HRVB, a deductive qualitative content analysis was conducted on qualitative study data. We focused on usability, acceptability, and perceived impact. Qualitative content analysis is an approach to qualitative data analysis that creates a concise description of a phenomenon from direct reports or documents (i.e., notes, videos, and other media) (Elo & Kyngäs, 2008). After multiple thorough readings of the study check-in notes and open-ended survey comments from 23 participants, initial content codes were developed by a study team member (GW). Coding was used to organize categories of shared experiences around barriers, facilitators, and impacts of HRVB. To improve rigor and ensure no categories or trends were missed, categories were evaluated by team members (MAC, RB, and CG) who had conducted participant meetings and were not involved in development of codes by validating categories and subcategories against raw data notes.

To address preliminary evidence of efficacy, we fit intent-to-treat (N = 28) linear mixed models with a random intercept and three repeated timepoints using SPSS v28. We used the restricted maximum likelihood estimator and an auto-regressive lag covariance structure. Estimated marginal means are shown for each timepoint and beta coefficients compared to baseline along with 95% confidence intervals, *p*-values, and effect sizes represented as Cohen’s *d*. Though we were not powered to detect the effects of adherence on changes in outcome, as a validation check, we ran a second set of models including adherence (as represented by days of HRVB practice ≥ 10 min) and the timepoint by adherence interaction to adjust the timepoint effects for levels of intervention engagement. Adherence data was available through the OptimalHRV web dashboard where an assigned group “administrator” may view study participants’ HRVB practice data and RMSSD for each baseline and post-intervention HRV reading. In terms of HRVB practice, we specifically collected session dates and times, (including minutes spent practicing), and subsequently downloaded the information into csv files for data analysis. Lastly, we conducted exploratory analyses of HRV change using mean RMSSD readings before and after completing the 2-month protocol using a paired *t*-test. We report Cohen’s *d* and absolute percent change.

## Results

### Feasibility

#### Recruitment and Enrollment

See Fig. 1 for a detailed flow of participants through phases of the study. Minimal challenges were encountered during recruitment and initial enrollment. In November 2021, we sent 185 emails (in 2 stages) advertising the study. After 34 days, we met our goal of 28 pre-enrolled participants (15%, 28/185 yield). Of the 28 devices mailed, all participants confirmed receipt and downloaded the study app. One participant formally withdrew from the protocol immediately after receiving the device and returned it to the study team. Within 2 months of the initial email invitations, 25 of the 28 pre-enrolled participants completed the app and device training. Thus, 89% of the consented sample qualified as fully enrolled and eligible to move forward with the study intervention.

#### Intervention Engagement

All 25 participants completed both prerequisite activities to begin HRVB training: (a) the RF assessment to establish the optimal breathing rate for HRVB practice and, (b) 5 baseline HRV readings. Mean number of participant check-in meetings was 6.8 (SD 2.9; range 0 to 11). One participant had no check-ins despite engaging in 46 days of ≥ 10 min of HRVB practice. Several participants preferred less frequent meetings and others met more often to accommodate varying circumstances.

Mean number of protocol days with at least 10 min of HRVB practice was 26.0 (SD 14.0; range 0 to 52). Of the 25 participants, 13 (52%) met the goal of at least 67% adherence (i.e., 28 or more days of HRVB practice). One participant had no HRVB practice due to problems connecting the device to the app, and 2 others logged fewer than 10 sessions—one of whom also had significant device operation problems and the other who temporarily lost the device during the trial. The remaining 22/25 (88%) participants had 14 days or more of ≥ 10 min of HRVB practice, representing a 33% or greater adherence rate; 40% of the sample (10/25) did at least 10 min of HRVB on more than 75% of protocol days. The mean minutes of total HRVB practice over the 2-month intervention was 494.0 (SD 286.0; range 0 to 1131).

#### Usability/Acceptability

The responses to the 5-point Likert scale questions are outlined in Table 2. Despite most of the sample noting device-to-app connection problems, most found HRVB useful and were likely to continue practicing after the study. We sent replacement devices to 4 participants; 2 of whom struggled with connecting the second device. Ultimately, 1 of these participants withdrew from the protocol; the other completed HRVB on 4 occasions and finished the post-intervention survey but not the post-intervention HRV readings.

**Table 2:** Acceptability and usability survey outcomes (N = 24)

Post-intervention survey question	n	%	Mean	SD	Median
Did you find the HRV biofeedback practice used for this study useful?					
Not at all useful (1)	0	0	3.88	1.15	4.00
Slightly useful (2)	4	17			
Moderately useful (3)	5	21			
Very useful (4)	5	21			
Extremely useful (5)	10	42			
How likely do you think you will continue to practice HRV biofeedback now that the study is over?					
Extremely unlikely (1)	0	0	4.08	0.97	4.00
Unlikely (2)	2	8			
Neither likely nor unlikely (3)	4	17			
Likely (4)	8	33			
Extremely likely (5)	10	42			
How likely are you to recommend HRV biofeedback to a friend or coworker?					
Extremely unlikely (1)	0	0	4.29	0.91	5.00
Unlikely (2)	1	4			
Neither likely nor unlikely (3)	4	17			
Likely (4)	6	25			
Extremely likely (5)	13	54			
Did you at any point during the study have device connectivity problems?					
Yes	19	79			
No	5	21			
To what extent did the device connectivity issues interfere with your ability to do biofeedback practices in accordance with the study protocol?^a^					
Never (1)	2	11	2	0.47	2.00
Sometimes (2)	15	79			
Half of the Time (3)	2	11			
Most of the Time (4)	0	0			
Always (5)	0	0			

^a^Only people who answered ‘Yes’ (n = 19) to the device connectivity problems were asked this question

##### Participant Experiences of Usability and Impact

The themes derived from the qualitative analyses are shown in Table 3. Common barriers to full engagement were busy schedules, fatigue, and technology difficulty; however, establishing a routine, focusing on how biofeedback may help one’s concerns, and the flexibility of the intervention all facilitated engagement. Participants felt that HRVB helped them to relax and improved their resilience during an extremely stressful time in their lives while also better connecting them to their body’s signals and experiences.

**Table 3:** Categories and subcategories from qualitative analysis of open-ended survey comments and check-in discussion notes (N = 23)

Category	Subcategory	Description	Supporting Data
Barriers to HRV	Tech Barriers (20/23)	Most participants had problems with the HRV app, sensor connectivity, or easily drained batteries. Many were resolved easily, but some interfered with ability to perform HRV and experience of the intervention	“The device connectivity issues in the beginning were frustrating but resolved fairly easily.” “Machine would not synch at times or not be recognized by iPhone delaying or omitting data.” Her biggest barrier this week was the device. She said she spoke with many different people on how to fix the device but was extremely frustrated
	Busy and Schedule (19/23)	Participants found it difficult to complete HRVB when they had many other things to do, especially work and family obligations. Changes in schedule were frequent disruptors of practice. This was pronounced, for some, when they practice HRVB for 20 min versus 10	“The only barriers were my schedule.” “Extremely busy work/school schedule.” She said that she feels that when she can find time in her day, it’s very useful, but that when her schedule changes a great deal, she can struggle to fit the biofeedback in His biggest barriers are being busy and his rotating schedule which makes it tough to establish a routine
	Tired and Sleepy (18/23)	Participants reported that being too tired after work or falling asleep made it difficult to complete HRVB	“The biggest barrier was that I would start to do the biofeedback and fall asleep doing it or get comfortable to do it and fall asleep before I even got to start the feedback exercise.” “It was easy to choose to skip it because I was tired.”
	Physical Aspects (5/23)	A small number of participants had difficulty participating in HRVB due to physiological barriers like breathing and pain using the sensor	“Inhaling and exhaling of the biofeedback was difficult because I couldn’t hold my breath for certain lengths of time.” The biggest problem she experiences is not being able to sync her breathing with the biofeedback because she has been ill. She said that she can’t breathe in deep enough and that she feels like she has to struggle to do even the quickest breathing rates She said that the finger sensor itself is painful. She began putting it on her middle finger instead of her pointer finger and that has been a bit less painful, but she noted that the sensor hurting her finger is “distracting” during biofeedback
Facilitators to HRV	Routine (12/23)	Participants who were able to integrate biofeedback into their routine reported that they were more successful at both daily practice and in working up to 20 min of daily practice	“I liked the sense of bedtime routine it provided.” She feels like she has her “system” which helps her be consistent She uses the reminders in the app to help with doing biofeedback consistently, but she says she has been remembering to do it even before her reminder lately She says she has felt the 20 min “flying by” and that she is not only feeling comfortable with the longer time but also “looking forward to it everyday.” She says she likes to have “20 min that’s mine” and plans to continue doing biofeedback after the study
	Helping Oneself (11/23)	Participants described how seeing benefits to themselves (or anticipating them) helped them to do HRV practice more regularly	“Biofeedback makes me feel nice and relaxed. I feel like there is something to look forward to relax me.” The biggest facilitator for her was feeling like she needed to keep doing well and that she felt that small moments of downtime were important for her and her mental health She said that it was almost to her advantage because she saw BF impact her positively because she so desperately needed stress relief, and she also felt motivated to continue because she needed relief from the panic at work
	Flexibility (8/23)	The flexibility of being able to achieve 20 min in one session or multiple sessions helped some participants achieve the goal	She says that she has no barriers to usage after being told that it’s okay to split her time into multiple sessions throughout the day She said she really likes doing the 20 min continuous as breaking up the times into 10-min increments is difficult as her busy day as a nurse She has been doing 20 min of biofeedback a day this week in broken up sessions throughout the day. She says she finds it easier to fit 20 min into her day when she breaks the time up He said splitting biofeedback up into two 10-min sessions has been more manageable for him
Impact of HRV	Calm and Relaxed (17/23)	Participants felt that HRVB gave them a general sense of calm, reduced anxiety, and improved sleep quality	Prior to beginning biofeedback, she was having a very difficult time sleeping and stated this is assisting her with sleep “I did it and then right after, I could just fall right asleep!” She has continued to be able to tell that she is feeling less stressed in her everyday life. She said she notices little things don’t bother her as much and she is calmer overall more consistently
	Resilience and Stress Relief (13/23)	Participants described feeling more able to handle stress due to biofeedback, both in general and by using breathing and mindfulness skills	“I have been able to control my breathing and use deep breathing during the day to help me calm and figure out what to do next in stressful situation.” “It takes the stress level down a notch.” “My stress levels have been the same as they always are, but I am able to cope with the stress. I have an improved ability to cope.” He says he has noticed he is less stressed and that he thinks it has helped him with his food cravings
	Mindfully Connecting with the Body (9/23)	Participants recognized the ways in which the app was strengthening connections between their emotions and their bodily sensations	“I look forward to the time and trying to predict numbers based on how I am feeling, sleeping and my general well-being.” “Not only has it helped me to calm my emotions and mentally step back, it has also opened my eyes to my body’s signals of stress and emotions.” “I try to spot check during the week to see how I am doing. I am definitely calmer, and more in sync with myself and my breathing.” He has enjoyed the longer 20-min sessions because he thinks they calm him down more and give him more time to be mindful about his breathing (making sure his exhale is longer than inhale, etc.)

### Preliminary Evidence of Efficacy

Mean pre-intervention HRV (reported as RMSSD) was 29.80 (SD = 13.93) and post-intervention was 33.66 (SD = 15.44) for the 23 completers, representing a 13% improvement, *t*(22) = 1.62, *p* = 0.120, Cohen’s *d* = 0.34.

Table 4 reports changes in scores pre- mid- and post-intervention for the 8 dimensions of interoceptive sensibility. Statistically significant early changes (i.e., by intervention mid-point) were seen on the following MAIA subscales: *noticing, attention regulation, self-regulation, body listening,* and *trusting* scores (*d*’s = 0.30—0.68; *p’s* < 0.032)*.* Between midpoint and post-intervention, scores on each of these dimensions further improved, with significant improvements for *attention regulation* (*d* = 0.53; *p* = 0.001)*, self-regulation* (*d* = 0.48; *p* = 0.008)*, and body listening* (*d* = 0.42; *p* = 0.014).

**Table 4:** Predicted means and mean changes from pre- to post-intervention for interoceptive sensibility outcomes derived from mixed models

Interoceptive Sensibility (MAIA subscales)	EMMs (SE)^a^	Betas of Mean change from Baseline (95% CI)	*p*-value	Cohen’s *d*
Noticing				
Pre-Intervention	2.97 (0.15)			
Mid-point	3.44 (0.16)	0.46 (0.20 to 0.73)	.001	0.59
Post -Intervention	3.52 (0.16)	0.55 (0.25 to 0.84)	.001	0.71
Not Distracting				
Pre-Intervention	1.52 (0.13)			
Mid-point	1.77 (0.13)	0.25 (− 0.10 to 0.60)	.200	0.38
Post -Intervention	1.82 (0.13)	0.30 (− 0.03 to 0.63)	.071	0.45
Not Worrying				
Pre-Intervention	2.62 (0.19)			
Mid-point	2.74 (0.20)	0.11 (− 0.23 to 0.46)	.510	0.12
Post -Intervention	3.12 (0.20)	0.50 (0.10 to 0.91)	.017	0.53
Attention Regulation				
Pre-Intervention	1.98 (0.18)			
Mid-point	2.60 (0.18)	0.62 (0.35 to 0.88)	< .001	0.68
Post -Intervention	3.08 (0.19)	1.10 (0.71 to 1.49)	< .001	1.20
Emotional Awareness				
Pre-Intervention	3.25 (0.17)			
Mid-point	3.49 (0.15)	0.24 (− 0.14 to 0.61)	.202	0.31
Post -Intervention	3.76 (0.14)	0.51 (0.16 to 0.87)	.005	0.65
Self-Regulation				
Pre-Intervention	2.40 (0.19)			
Mid-point	2.94 (0.20)	0.54 (0.21 to 0.87)	.002	0.55
Post -Intervention	3.41 (0.20)	1.01 (0.61 to 1.41)	< .001	1.02
Body Listening				
Pre-Intervention	1.69 (0.23)			
Mid-point	2.39 (0.24)	0.70 (0.32 to 1.09)	.001	0.59
Post -Intervention	2.89 (0.24)	1.20 (0.76 to 1.64)	< .001	1.02
Trusting				
Pre-Intervention	2.81 (0.25)			
Mid-point	3.18 (0.26)	0.37 (0.03 to 0.71)	.032	0.30
Post-Intervention	3.39 (0.26)	0.58 (0.24 to 0.91)	.002	0.46

Baseline N = 28; mid and post-intervention N = 24

^a^Estimated marginal means with robust standard errors

Table 5 presents changes in disordered eating, intuitive eating, body appreciation, perceived stress, resilience, and mindful self-care scores. Early changes were evident in EDE-Q7 and LOCES scores, as well as intuitive eating, body appreciation, and mindful self-care (*d*’s = 0.37—0.63; *p*’s < 0.005). Further significant improvements were seen in LOCES scores between mid and post-intervention (*d* = 0.30; *p* = 0.021) and mindful self-care (*d* = 0.34; *p* = 0.010); EDE-Q7 scores continued to improve, but mid-to-post change did not reach statistical significance (*d* = 0.23; *p* = 0.106). Overall, except for the *not distracting* dimension of interoceptive sensibility, all outcomes showed significant improvements when comparing baseline to post intervention (see Table 5).

**Table 5:** Predicted means and changes from pre- to post-intervention for disordered eating, stress, resilience, and mindful self-care outcomes derived from mixed models

Variable	EMMs (SE)^a^	Betas of Mean change from Baseline (95% CI)	*p*-value	Cohen’s *d*
Global Disordered Eating				
Pre-Intervention	4.07 (0.25)			
Mid-point	3.47 (0.26)	− 0.60 (− 0.97 to − 0.23)	.002	0.45
Post-Intervention	3.17 (0.26)	− 0.90 (− 1.29 to − 0.51)	< .001	0.68
Loss of Control Eating				
Pre-Intervention	3.43 (0.17)			
Mid-point	2.94 (0.18)	− 0.49 (− 0.72 to − 0.26)	< .001	0.54
Post-Intervention	2.67 (0.18)	− 0.76 (− 1.05 to − 0.47)	< .001	0.83
Intuitive Eating				
Pre-Intervention	2.38 (0.15)			
Mid-point	2.87 (0.15)	0.49 (0.27 to 0.71)	< .001	0.62
Post-Intervention	2.88 (0.15)	0.50 (0.22 to 0.78)	.001	0.63
Body Appreciation				
Pre-Intervention	2.73 (0.16)			
Mid-point	3.03 (0.16)	0.30 (0.10 to 0.50)	.005	0.37
Post-Intervention	3.14 (0.16)	0.41 (0.18 to 0.65)	.001	0.50
Perceived Stress				
Pre-Intervention	21.07 (1.03)			
Mid-point	18.90 (1.09)	− 2.18 (− 4.43 to 0.08)	.058	0.40
Post-Intervention	14.46 (1.08)	− 6.62 (− 8.69 to − 4.53)	< .001	1.22
Sense of Coherence				
Pre-Intervention	46.32 (1.18)			
Mid-point	47.57 (1.25)	1.25 (− 1.17 to 3.67)	.301	0.21
Post-Intervention	49.07 (1.25)	2.75 (0.30 to 5.21)	.030	0.45
Mindful Self-Care				
Pre-Intervention	2.84 (0.11)			
Mid-point	3.22 (0.12)	0.38 (0.23 to 0.54)	< .001	0.63
Post-Intervention	3.42 (0.12)	0.59 (0.42 to 0.76)	< .001	0.98

N = 28 at pre-intervention, N = 24 at midpoint; N = 24 at post intervention

^a^Estimated Marginal Means with Robust Standard Errors

Given lack of a control arm, we adjusted for the number of HRVB practice days on the mean changes over time as a validation check. Detailed results are shown in Supplementary Tables B and C. While intervention adherence did not predict changes in outcome for any model (likely due to inadequate power), there were significant main effects for days of HRVB practice on *not worrying* (*b* = 0.023; SE = 0.012; *p* = 0.049) and *attention regulation* (*b* = 0.028; SE = 0.010; *p* = 0.011). Additionally, controlling for adherence reduced the effect sizes of time for several outcomes (e.g., global disordered eating, *body listening,* and *trusting*) suggesting that adherence partially mediated change in these scores. However, in other outcomes, the effects of time were even stronger after controlling for adherence (e.g., loss of control eating, *self-regulation, noticing*, body appreciation, and perceived stress), suggesting adherence may operate as a suppressor effect for these scores. Outcomes that maintained significant (or marginally significant) changes between baseline and post-intervention after controlling for adherence effects on change included: *noticing* (*d* = 0.90; *p* = 0.024), *attention regulation* (*d* = 1.38; *p* = 0.001), *self-regulation* (*d* = *1.48; p* < 0.001), *body listening* (*d* = *0.77; p* = 0.064), resilience (*d* = *0.82; p* = 0.088), perceived stress (*d* = *1.42; p* = 0.002), mindful self-care (*d* = *1.01; p* = 0.002), body appreciation (*d* = *0.96; p* = 0.005), intuitive eating (*d* = *0.72; p* = 0.050), LOCES (*d* = *1.15; p* = 0.002), and EDE-Q7 scores (*d* = *0.61; p* = 0.088). Outcomes that did not show improvements once controlling for the impact of intervention adherence on changes over time included *not distracting, not worrying, emotional awareness, and trusting* (*p’s* > 0.142).

## Discussion

This study adds to the growing literature base showing a potential place for the use of mindfulness-based smartphone apps to improve well-being in stressed populations (Schwartz et al., 2023). The mixed methods feasibility design of the trial tested implementation, recruitment, retention, engagement, acceptability, and early signals of efficacy of an HRVB app for healthcare providers coping with disordered eating during the COVID-19 pandemic. Recruitment was a success, with implementation strategies automating many components of the study enrollment process to reduce no-shows on consent interviews (pre-enrollment) and device/app training meetings (full enrollment). Despite difficulties with the device-to-app connectivity and significant time barriers to practicing HRVB, 75% (18/24) of the sample intended to continue using the intervention and 79% (19/24) would be very or extremely likely to recommend it to others. Therefore, this study provides encouraging feasibility evidence that the tool is a potentially useful and acceptable coping strategy for healthcare workers. In light of the distress and burnout reported in this population (Copel et al., 2023; Heavner et al., 2023), a usable, low-cost simple intervention like app-based HRVB that can be performed essentially anywhere could be a vital tool to diffuse the inevitable stressors confronted by healthcare workers throughout their workday.

A recent meta-analysis found that attrition rates in smartphone app intervention studies are a significant concern (Meyerowitz-Katz et al., 2020). A large scale translational study also identified that less than 3% of people who downloaded an app to improve eating continued to use the app regularly (Helander et al., 2014). Meyerowitz-Katz et al.’s (2020) meta-analysis reported the pooled dropout rate across 17 app-based studies was 43% overall. The low dropout rate in our study (14%, 4/28) speaks to the potential for OptimalHRV as an mHealth intervention.

In terms of engagement, our intervention performed comparatively or better than similar studies utilizing mindfulness-based apps (Pratt et al., 2023; Schuman et al., 2023). While we failed to reach our goal of 75% participants doing meaningful HRVB on *most* days (≥ 67%), 88% engaged in HRVB on 33% or more of the protocol days. Moreover, 40% of the sample did meaningful HRVB on 75% or more of the days. This is favorable compared to another recent feasibility study of a mindfulness-based app for nurses during the pandemic where 19% (13/69) of the sample completed 75% or more of the daily sessions (Pratt et al., 2023). HRVB and other mindfulness-based apps may have great promise, but uptake is going to take strategic advancements on user experience to fully realize the potential health enhancing features of these technologies.

One difficulty with improving the high attrition rates found in research on app-based interventions for health is the paucity of studies reporting detailed attrition outcomes. Even for the studies that do provide attrition information, few examine the drivers of attrition. One strength of the present study is the ability to triangulate knowledge from our mixed methods of data collection. We know that device-to-app connectivity was a factor in use levels at least some of the time for nearly 80% of the sample. We also know that overall fatigue and lack of time were the most common barriers to engagement, which is not surprising given the context of the study. To prevent a lost opportunity to explore potential explanations for intervention engagement, we ran exploratory post hoc correlations between days of HRVB practice and 10 baseline factors available from the CHAMPS Registry (Kaufmann et al., 2021) in which the participants had enrolled the year prior (see Supplementary Table D).

Given the small sample size, we only considered effect sizes greater than *r* = 0.30 as a potential signal of attrition risk to examine in future studies. Age and burnout met this criterion (*r* = – 0.35 and *r* = – 0.32, respectively). Older individuals and those with higher scores on the Oldenburg Burnout Inventory (Demerouti et al., 2001) tended to have lower adherence. These associations might suggest that comfort level with technology for older generations and job burnout could potentially inhibit uptake of such interventions. Though the relationships are post hoc in nature, they are consistent with the barriers found in the qualitative comments.

With critical markers of feasibility established in the present research, an important next step will be to test this app in a larger scale setting. In addition, this study supported early evidence for potential efficacy of HRVB using this smartphone app for improving disordered eating outcomes, mindful self-care behaviors (including intuitive eating and body appreciation), and several dimensions of interoceptive sensibility, especially attention regulation and self-regulation. The intervention also showed improvements in stress and though to a lesser extent, resilience. Even after accounting for the impact of adherence, changes in attention and self-regulation were very large as were changes in mindful self-care behaviors, loss of control eating, and stress, with effect sizes greater than 1. These findings contribute to existing evidence of efficacy for multiple well-being outcomes among a similar sample of healthcare workers tested using a mobile HRVB intervention pre-pandemic (Buchanan & Reilly, 2019).

Though replication in a larger, controlled trial is necessary, our efficacy findings are consistent with results from a recent randomized control trial showing a mindfulness-based app HeadSpace (n = 1095) was superior against an active control (n = 1087) for reducing stress and improving well-being in healthcare workers (Taylor et al., 2022). In the HeadSpace trial, analyses showed 2 significant mechanisms explained reduction in stress over time—formal engagement with the intervention and self-compassion. Considering that the mindful self-care scale adopted for the present study contained multiple self-compassion items, future research should consider it as a potential mediator of the positive changes in disordered eating, especially given the sizable and *early* effects of HRVB on mindful self-care.

Similarly, in a model of stress among healthcare workers, Hotchkiss found mindful self-care mediated the relationship between lower compassion satisfaction and burnout (Hotchkiss, 2018). This provides further evidence for investigating mindful self-care as a potential mechanism of change in coping. Of note, we also saw significant improvements at midpoint in appreciating the body and eating intuitively, both unique aspects of mindful self-care. Finally, significant early changes were reported in dimensions of interoceptive sensibility, such as listening to and trusting the body, as well as the capacity to regulate attention and distress by noticing bodily cues. Thus, investigations of interoception as mediators of later change in disordered eating are another future research direction. Testing for mechanisms would offer more information about processes underlying this new potentially effective tool and reveal whether the OptimalHRV app operates similarly to HeadSpace, an alternative mindfulness-based app with convincing effectiveness data (Taylor et al., 2022).

### Strengths and Limitations

The present study is strengthened by the abundance of formative usability data (qualitative and quantitative) and outcomes (e.g., disordered eating, body appreciation, and intuitive eating) about which we lack information surrounding the use of HRVB. We also have a wide range of demographic representation with participants from varying regions of the US. However, there are important limitations to be acknowledged. Specifically, efficacy findings must be viewed as only preliminary given the lack of a control arm and small sample. Moreover, technical issues with the sensors and app may have contributed to impaired participation and/or our ability to track use of HRVB practices. Participants also may have engaged in HRV breathing practices outside of the app which were not captured in our data. Though the sample consisted of a diverse cross-section of healthcare occupations, it was composed mostly of nurses working at metropolitan hospitals who were volunteers willing to be in a study. Therefore, our results may not generalize to a randomly identified group of healthcare workers. We recruited few males and people of color into the study. Use of HRVB in racial and ethnic minority groups is an especially important direction for future research. Lastly, given the study took place during the COVID-19 pandemic, several participants or their close family members had COVID-19 during study participation, potentially impacting HRV readings and feasibility evaluations.

### Concluding Remarks

In the early stages of the COVID-19 pandemic, Aristizabal et al. (2020) considered HRVB an ideal intervention for enhancing resilience in frontline workers dealing with elevated distress and burnout. This study provided feasibility data and encouraging preliminary evidence to support this claim and had the additional benefit of informing healthcare professionals about new mind–body coping strategies and reducing maladaptive eating. Research shows that long hours, stress, and fatigue reported by healthcare workers present barriers to self-care, specifically eating nourishing meals (Marko et al., 2022) which is a common precursor to symptoms of loss of control eating as reported in the sample participating in this study. It is reassuring to see that a low-cost and convenient tool like doing HRVB practice on an app was associated with such broad-based positive outcomes. Our qualitative data reinforced confidence in the signals of efficacy seen by supporting a recognition that HRVB afforded a general sense of calmness. The app evaluated in this study warrants further investigation as a potentially promising method for buffering against work-related stressors in healthcare professionals through connecting to their body and mind in a different way.

### Supplementary Information

Below is the link to the electronic supplementary material.

Supplementary file1 (DOCX 27 KB)

## Data Availability

The data that support the findings of this study are available upon request from the corresponding author at jmensing@nova.edu.
