# Supplementary material for: A Pilot Feasibility Evaluation of a Heart Rate Variability Biofeedback App to Improve Self-Care in COVID-19 Healthcare Workers
Source: Appl Psychophysiol Biofeedback. 2024 Mar 19;49(2):241–59. doi: 10.1007/s10484-024-09621-w (PMC11101559; doi:10.1007/s10484-024-09621-w)
Supplement: Supplementary file 1 — Supplementary file1 (DOCX 27 KB) [file 10484_2024_9621_MOESM1_ESM.docx]

| **Supplementary Table A.** *Modifications to the Brief Mindful Self-Care Scale by Hotchkiss and Cook-Cottone* | | | |
| --- | --- | --- | --- |
| **Subscale name** | **Original Item** | **Modified item** |  |
| Physical care | “I practiced yoga or another mind/body practice (e.g., Tae Kwon Do, Tai Chi)” | “I practiced yoga or another mind/body practice (e.g., Biofeedback, Tae Kwon Do, Tai Chi)” |  |
| Supportive Structure | “I kept my work/schoolwork area organized to support my work/schoolwork tasks” | “I kept my work/schoolwork/living area organized to support my daily tasks” |  |
| Self-compassion and Purpose | “I experienced meaning and/or a larger purpose in my work/school (e.g., for a cause)” | “I experienced meaning and/or a larger purpose in my life (e.g., living for a cause)” |  |

| **Supplementary Table B.** *Predicted means and changes from pre- to post-intervention for interoceptive sensibility outcomes adjusted for effects of adherence* | | | | |
| --- | --- | --- | --- | --- |
| **Interoceptive Sensibility (MAIA subscales)** | **EMMs (SE)**^a^ | **Betas of Mean change from Baseline (95% CI)** | ***p*-value** | **Cohen's *d*** |
| Noticing |  |  |  |  |
| Pre-Intervention | 2.99 (0.16) |  |  |  |
| Mid-point | 3.46 (0.17) | 0.69 (0.03 to 1.35) | .041 | 0.89 |
| Post-Intervention | 3.54 (0.17) | 0.70 (0.10 to 1.31) | .024 | 0.90 |
|  |  |  |  |  |
| Not Distracting |  |  |  |  |
| Pre-Intervention | 1.55 (0.13) |  |  |  |
| Mid-point | 1.78 (0.14) | 0.46 (-0.34 to 1.26) | .254 | 0.70 |
| Post-Intervention | 1.82 (0.14) | 0.33 (-0.37 to 1.04) | .348 | 0.50 |
|  |  |  |  |  |
| Not Worrying |  |  |  |  |
| Pre-Intervention | 2.68 (0.18) |  |  |  |
| Mid-point | 2.76 (0.19) | 0.15 (-0.64 to 0.95) | .694 | 0.16 |
| Post-Intervention | 3.14 (0.19) | 0.53 (-0.32 to 1.39) | .213 | 0.56 |
|  |  |  |  |  |
| Attention Regulation |  |  |  |  |
| Pre-Intervention | 2.05 (0.17) |  |  |  |
| Mid-point | 2.63 (0.17) | 0.39 (-0.26 to 1.05) | <.226 | 0.43 |
| Post-Intervention | 3.10 (0.18) | 1.26 (0.53 to 1.99) | .001 | 1.38 |
|  |  |  |  |  |
| Emotional Awareness |  |  |  |  |
| Pre-Intervention | 3.27 (0.18) |  |  |  |
| Mid-point | 3.47 (0.15) | -0.12 (-0.81 to 0.57) | .715 | 0.15 |
| Post-Intervention | 3.77 (0.15) | 0.60 (-0.21 to 1.40) | .142 | 0.76 |
|  |  |  |  |  |
| Self-Regulation |  |  |  |  |
| Pre-Intervention | 2.47 (0.18) |  |  |  |
| Mid-point | 3.00 (0.19) | 0.99 (0.16 to 1.81) | .021 | 1.00 |
| Post-Intervention | 3.46 (0.19) | 1.46 (0.71 to 2.12) | <.001 | 1.48 |
|  |  |  |  |  |
| Body Listening |  |  |  |  |
| Pre-Intervention | 1.73 (0.23) |  |  |  |
| Mid-point | 2.40 (0.24) | 0.48 (-0.35 to 1.31) | .245 | 0.41 |
| Post-Intervention | 2.89 (0.24) | 0.91 (-0.06 to 1.88) | .064 | 0.77 |
|  |  |  |  |  |
| Trusting |  |  |  |  |
| Pre-Intervention | 2.83 (0.26) |  |  |  |
| Mid-point | 3.16 (0.27) | -0.23 (-0.98 to 0.52) | .533 | 0.18 |
| Post-Intervention | 3.37 (0.26) | 0.10 (-0.63 to 0.85) | .767 | 0.08 |
| *Notes.* Baseline N=28; mid and post-intervention N=24; ^a^Estimated Marginal Means, Robust Standard Errors, Betas weights and 95% CIs derived from Linear Mixed Models controlling for adherence and the interaction effect between time and adherence | | | | |

| **Supplementary Table C.** *Predicted means and changes from pre- to post-intervention for disordered eating, stress, resilience, and mindful self-care outcomes adjusted for effects of adherence* | | | | | |
| --- | --- | --- | --- | --- | --- |
| **Variable** | **EMMs (SE)**^a^ | | **Betas of Mean change from Baseline (95% CI)** | ***p*-value** | **Cohen's *d*** |
| Global Disordered Eating | |  |  |  |  |
| Pre-Intervention | | 4.04 (0.26) |  |  |  |
| Mid-point | | 3.46 (0.31) | -0.49 (-1.21 to 0.24) | .177 | 0.37 |
| Post-Intervention | | 3.15 (0.26) | -0.81 (-1.74 to 0.13) | .088 | 0.61 |
|  | |  |  |  |  |
| Loss of Control Eating | |  |  |  |  |
| Pre-Intervention | | 3.41 (0.18) |  |  |  |
| Mid-point | | 2.91 (0.18) | -0.81 (-1.30 to -0.31) | <.003 | 0.89 |
| Post-Intervention | | 2.64 (0.18) | -1.05 (-1.66 to -0.43) | <.002 | 1.15 |
|  | |  |  |  |  |
| Intuitive Eating | |  |  |  |  |
| Pre-Intervention | | 2.40 (0.15) |  |  |  |
| Mid-point | | 2.87 (0.15) | 0.36 (-0.15 to 0.87) | .165 | 0.46 |
| Post-Intervention | | 2.89 (0.16) | 0.57 (0.00 to 1.14) | .050 | 0.72 |
|  | |  |  |  |  |
| Body Appreciation | |  |  |  |  |
| Pre-Intervention | | 2.77 (0.16) |  |  |  |
| Mid-point | | 3.06 (0.16) | 0.37 (-0.02 to 0.77) | .062 | 0.45 |
| Post-Intervention | | 3.19 (0.26) | 0.78 (0.26 to 1.30) | .005 | 0.96 |
|  | |  |  |  |  |
| Perceived Stress | |  |  |  |  |
| Pre-Intervention | | 20.86 (1.05) |  |  |  |
| Mid-point | | 18.81 (1.11) | -2.97 (-8.10 to 2.17) | .248 | 0.55 |
| Post-Intervention | | 14.33 (1.10) | -7.72 (-12.51 to -2.92) | .002 | 1.42 |
|  | |  |  |  |  |
| Sense of Coherence | |  |  |  |  |
| Pre-Intervention | | 46.27 (1.21) |  |  |  |
| Mid-point | | 47.51 (1.27) | 0.25 (-4.58 to 5.09) | .916 | 0.04 |
| Post-Intervention | | 49.17 (1.27) | 4.97 (-0.77 to 10.72) | .088 | 0.82 |
|  | |  |  |  |  |
| Mindful Self-Care | |  |  |  |  |
| Pre-Intervention | | 2.87 (0.11) |  |  |  |
| Mid-point | | 3.24 (0.12) | 0.41 (0.04 to 0.78) | .030 | 0.68 |
| Post-Intervention | | 3.45 (0.12) | 0.61 (0.25 to 0.98) | .002 | 1.01 |
| *Notes.* Baseline N=28; mid and post-intervention N=24; ^a^Estimated Marginal Means, Robust Standard Errors, Betas weights, and 95% CIs derived from Linear Mixed Models controlling for adherence and the interaction effect between time and adherence | | | | | |

| **Supplementary Table D.** *Post hoc correlation coefficients with adherence to the intervention measured in days practiced at least 10 minutes of HRV biofeedback* (N=28) | | |
| --- | --- | --- |
| **Variable** | **Pearson *r*** | ***p*-value** |
| 1. Age (in years) | **-0.348** | .069 |
| 2. Years in profession^a^ | -0.250 | .208 |
| 3. Resilience (Mean Brief Resilience Scale) | 0.260 | .182 |
| 4. Burnout (Sum of Oldenburg Burnout Inventory) | **-0.315** | .102 |
| 5. Anxiety (Sum of Generalized Anxiety Disorder-7) | 0.048 | .808 |
| 6. Depression (Sum of Patient Health Questionnaire-2) | 0.070 | .725 |
| 7. Trauma (Sum of Impact of Experiences Scale-Revised) | 0.125 | .527 |
| 8. Social Support (Sum of Oslo Social Support Scale) | 0.147 | .456 |
| 9. Insomnia (Sum of Insomnia Severity Index) | 0.052 | .791 |
| 10. Proactive Coping (Sum of Proactive Coping subscale^b^) | 0.027 | .893 |
| *Notes.* ^a^This variable had one missing response (N=27); ^b^from the Proactive Coping Inventory; Bold indicates a moderate effect size and potentially meaningful correlation | | |
